# Course of psychological symptoms and the initial management strategies in general practice using electronic health records in the Netherlands

**DOI:** 10.1136/bmjopen-2025-108541

**Published:** 2026-04-21

**Authors:** Asma Chaabouni, Barbara Schouten, Juul Houwen, Peter Lucassen, Reinier Akkermans, Iris Walraven, Kees van Boven, Henk Schers, Tim Olde Hartman

**Affiliations:** 1 Department of Primary and Community Care, Radboud Institute of Medical Innovation, Radboud University Medical Centre, Radboudumc, Nijmegen, the Netherlands; 2 IQ Health Science Department, Radboud Institute of Medical Innovation, Radboud University Medical Centre, Radboudumc, Nijmegen, the Netherlands

**Keywords:** Primary Health Care, EPIDEMIOLOGY, General Practice

## Abstract

**Abstract:**

**Background:**

Patients who consult their GP about psychological complaints, such as feeling anxious or depressed, are often initially given a psychological symptom diagnosis. However, it remains unclear whether these symptoms will develop into psychiatric conditions, which is crucial for informing patients about their prognosis and guiding GPs in their management.

**Aims:**

To explore the course of psychological symptom diagnoses and compare GPs’ management strategies for (a) psychological symptom diagnoses that persisted for more than a year and (b) those that changed into psychiatric conditions during the first year of care.

**Methods:**

We performed a retrospective cohort study using the Family-Medicine Network database. We included all episodes of care (EoC) that started with a psychological symptom diagnosis between 2008 and 2021. We performed negative binomial analyses and logistic regression analyses to compare management strategies and number of contacts between EoC that changed into psychiatric conditions and persistent psychological symptoms (>1 year) during the first year of care.

**Results:**

Out of the 14 633 EoC that started with a psychological symptom diagnosis, 79.4% resolved within 1 year, 12.8% persisted as psychological symptoms and 7.8% changed into psychiatric conditions. In EoC that changed into psychiatric conditions, as compared with EoC that persisted as psychological symptoms, we observed a significantly higher number of contacts with the GP (RR=1.76, 95% CI 1.63 to 1.91) as well as an increased total number of interventions (RR=1.71, 95% CI 1.58 to 1.84).

**Conclusion:**

Most psychological symptoms remain for only a short period of time and only a few persist or change into psychiatric conditions. Future research should investigate the factors that influence patients’ decisions to seek further help from their GPs as well as those that contribute to the transition from psychological symptom diagnoses to a psychiatric condition.

**STRENGTHS AND LIMITATIONS OF THIS STUDY**

- The study uses large, longitudinal and high-quality primary care registration data from the Family-Medicine Network registry covering over a decade of real-world episodes of care (EoC).
- EoC was defined and structured using International Classification of Primary Care coding, ensuring consistency in recording diagnoses, interventions and contacts.
- Detailed data on referrals and pharmacological treatment were available, but individual patient socioeconomic status and context variables were limited.
- Diagnostic coding does not capture the severity or complexity of underlying health problems, factors that may have influenced the course of symptoms.

## Introduction

 Patients frequently consult their General Practitioners (GPs) about psychological complaints, such as feeling anxious or depressed. During the consultation, the GP may explore the underlying reasons for these complaints, assess additional symptoms and determine whether the patient’s presentation meets the criteria for a psychiatric disorder (eg, depressive or anxiety disorders). When the symptoms do not fulfil the criteria for a psychiatric disorder, GPs usually label the patient’s main complaint as a symptom diagnosis, such as ‘feeling depressed’.^1^,^4^ In primary care, symptom diagnoses are a commonly used diagnostic category to describe symptoms that do not fulfil the criteria for a disease or disorder. In these circumstances, a symptom diagnosis represents the most appropriate medical label for the health problem.^4^ During the follow-up of patients with a psychological symptom diagnosis, symptoms may resolve, change into a psychiatric condition or persist as a psychological symptom diagnosis.

The occurrence of symptom diagnoses in general practice is high. A Danish study showed that out of all consultations with patients presenting a health problem, the GP made a symptom diagnosis in 36% of the cases.^5^ A Dutch study showed a higher prevalence (58%) of symptom diagnoses.^6^ However, these studies only focused on somatic symptom diagnoses, with a lack of information about the course and management of psychological symptom diagnoses. This is particularly important in the context of general practice, where consultations for mental health problems are more demanding and tend to take longer.^7^ Management of patients with psychological symptoms may be especially complex because these patients tend to also have more somatic symptoms compared with patients without psychological symptoms.^8^ The lack of knowledge about the course and management of psychological symptom diagnoses is problematic as this knowledge could facilitate informing patients and improving the prognosis of their psychological symptoms.

Through this exploratory study, we first aim to gain an initial understanding and better insight into the course of psychological symptoms in general practice. Second, we aim to compare management strategies during the first year between psychological symptoms that changed into psychiatric conditions and psychological symptoms that persisted over a year.

## Methods

### Design

We performed a retrospective cohort study in a practice-based registration network (PBRN) namely the Family-Medicine Network (FaMe-Net) database. The current study was pre-registered (https://doi.org/10.17605/OSF.IO/4JMNU) and has been performed according to open science principles. The current study is part of the training network Encompassing Training in Functional Disorders across Europe; https://etude-itn.eu/, a network that aims to improve the understanding of mechanisms, diagnosis, treatment and stigmatisation of functional disorders.^9^ Data analysed in this study are pseudonymised.

### Setting

FaMe-Net is a primary care registration network using electronic health records that currently includes over 40 000 patients registered in six general practices from the regions of Nijmegen and Amsterdam in the Netherlands.^10^ The FaMe-Net population is representative of the Dutch population in terms of age, sex and social class.^11^ To improve the reliability of the registration, the FaMe-Net GPs have regular training opportunities, including meetings to discuss the coding system and biannual online ‘uniformity’ surveys are to promote consistency through training.^10^ In the Netherlands, GPs act as gatekeepers, and all residents are registered with a single GP practice.

The GPs participating in the network use the International Classification of Primary Care (ICPC-2) to code all contacts with patients. ICPC-2 is divided into 17 different chapters:^12 13^ 15 chapters on somatic problems, one chapter on psychological problems (P) and one chapter on social problems (Z).^12 13^ Each chapter consists of seven different components: (1) Symptoms and complaints, (2) Diagnostic, screening and preventative procedures, (3) Medication, treatment and procedures, (4) Test results, (5) Administrative procedures, (6) Referrals and (7) Diseases.^14^

All contacts in the FaMe-Net database are registered within an episode of care (EoC) structure, defined as a health problem in a patient from the first until the last contact.^10^ The EoC includes information about the reason for encounter, the diagnosis and the process. The diagnosis is made by the GP and can be modified during consecutive contacts within the episode. As such, the diagnosis could change from “feeling anxious’’ to “anxiety disorder’’ over the contacts if the patient gradually fulfils the diagnostic criteria of an “anxiety disorder’’. The database also contains coded information about the process defined as the actions undertaken by the GP: interventions, referrals and treatment including medication.^10^ Medication is coded following the Anatomical Therapeutic Chemical Classification and linked to the EoC. Within FaMe-Net, the EoC structure enables longitudinal tracking of patient information. Changes such as a patient being seen by a different GP within the same practice or experiencing long-term hospitalisation are recorded within the Dutch healthcare system and are, in principle, available to GPs and integrated into the registration system.

During a single medical contact between a patient and a GP, it is possible to record more than one EoC, depending on the patient’s needs and requests for care during that single medical contact. EoC diagnoses are coded and updated based on the highest level of diagnostic certainty during medical contacts. Within the FaMe-Net system, multiple EoCs can be active simultaneously. As a result, the course and management of a symptom diagnosis may occur in the context of other ongoing conditions. Importantly, GPs register and update diagnoses within each EoC based on their overall clinical assessment, which includes knowledge of comorbidities and intercurrent illnesses. While EoCs are analytically separated, the diagnostic and management decisions recorded within them are made in the context of the patient’s full clinical picture.

**Figure 1:**
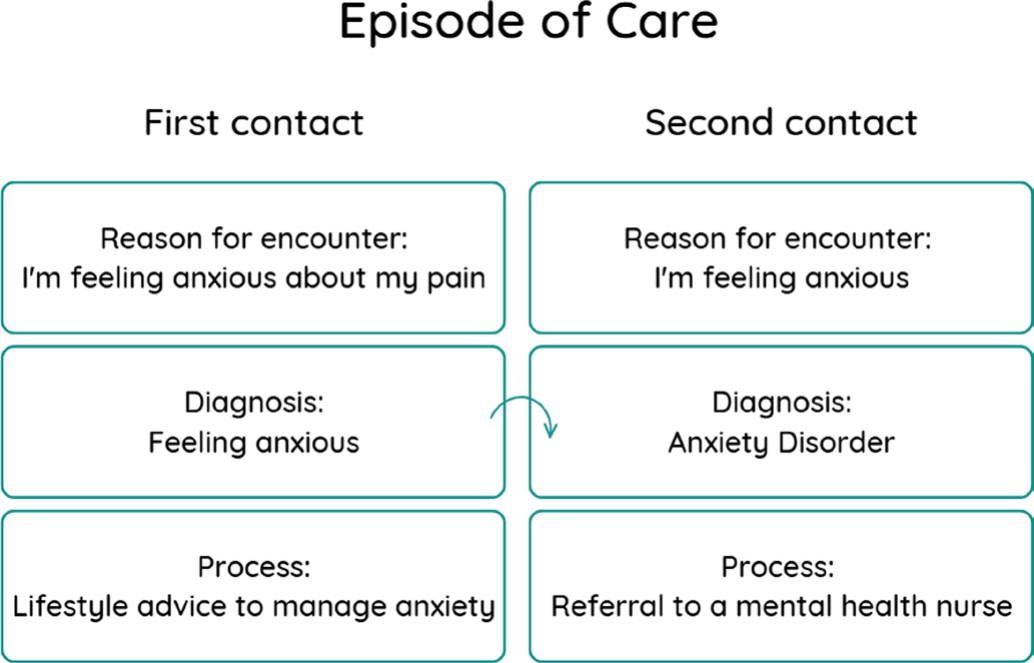
An Episode of Care structure consisting of two contacts.

The flow chart below (figure 1) illustrates an example of an EoC structure consisting of two contacts. It illustrates an example of an EoC for anxiety disorder as a final diagnosis. It begins with the initial complaint, “I feel anxious”, which is initially recorded as a symptom diagnosis. In a subsequent contact, the diagnosis is updated to an anxiety disorder as the patient’s anxiety symptoms now meet the criteria for an anxiety disorder based on the GP’s interpretation.

### Data collection

#### Demographics

We collected patients’ characteristics such as age at the first contact date of the EoC and sex. We excluded children and adolescents aged below 18 because this age group has a different pattern and range of psychological symptoms. More socioeconomic variables were available through forms filled in by patients. However, these data were available for only approximately 20% of the study population and were considered insufficiently representative to be included in the main analyses. Exploratory analyses showed that patients with available SES data differed systematically from those without these data (eg, they were significantly younger), suggesting potential selection bias. Therefore, these variables were not incorporated into the analyses.

#### Episodes of care

We selected all EoC that started with a psychological symptom diagnosis during the study period between 2008 and 2021 (see online supplemental appendix 1 included ICPC-2 codes). In order to have a follow-up period of at least 1 year in all EoC, we followed the course of EoC that started in 2021 over the year 2022. We excluded EoC that started with behaviour problems, substance use and phase of life problems. Phase of life problems include empty nest syndrome, mid-life crisis and retirement problems.

We defined the episode duration as the time between the first and the last contact for each EoC. Then, we identified four distinct patterns in the evolution of a symptom diagnosis: (1) The psychiatric condition group for episodes that started as a psychological symptom diagnosis and changed into a psychiatric condition at some point during the EoC (psychiatric group), (2) The persistent psychological symptom diagnosis group for episodes in which the psychological symptom persisted for more than a year (persistent group), (3) The transient psychological symptom diagnosis group for psychological symptoms that resolved spontaneously within a year (transient group) and (4) The “other health problems’’ group for episodes that started with psychological symptoms and changed into other conditions such as somatic conditions or social problems (other group). If the EoC didn’t change into a psychiatric condition at any point or any other health problem as the last diagnosis, we classified the EoC as a psychological symptom diagnosis. When that EoC lasted longer than a year, we classified it as a persistent psychological symptom diagnosis. For EoC classified as transient, the term “resolved” does not imply confirmed clinical recovery. Rather, it indicates the patient did not seek further help from their GP for that problem. To visualise the course of psychological symptoms, we used alluvial diagrams. These diagrams show how the diagnoses of psychological symptoms changed at different time points of each EoC, such as the first and the last contacts.

#### Number of contacts

We counted the number of contacts during each EoC for face-to-face consultations, out-of-hours services, telephone and e-consultations.

#### Management strategies

We counted the total number of interventions (see online supplemental appendix 2 for the types of interventions) as well as medication and referrals during each episode. Concerning medication, we specifically focused on prescriptions relevant to psychological problems (ie, antidepressants, anxiolytics, antipsychotics, benzodiazepines and beta-blockers). Concerning referrals, we counted the total number of referrals to primary and secondary care in general, then specified the number of referrals relevant to psychological symptom diagnoses such as the number of referrals to primary care psychologists, psychotherapists, psychiatrists and mental health institutions, medical specialists and mental health institutions.

### Data analysis

We performed descriptive statistics to summarise data about the course of psychological EoC, demographics, numbers of contact and management strategies during the whole EoC duration with measures of centrality and measures of dispersion. We determined mean and SD or median and 25%–75% percentiles for continuous variables, and frequencies and percentages for categorical variables.

We explored differences in the management strategies of GPs during the first year of the follow-up period between episodes of the persistent and psychiatric groups. Therefore, we compared the GP’s management strategies and number of contacts between these courses using T-tests for normally distributed continuous variables and negative binomial regression analysis for count variables. In addition, we used χ^2^ or binary logistic regression for dichotomous variables. Rate ratios (RRs) are reported for count variables and ORs are reported for categorical dichotomous variables (OR).

A p value of <0.05, based on two-sided testing, was considered as statistically significant. Statistical analyses were performed using the Statistical Package for Social Science (SPSS) V.29.0.0.

### Patient and public involvement

None.

## Results

Between 2008 and 2021, we identified a median number of patients of 30 203 consulting for 1 209 681 EoC. Of all these episodes, 14 633 episodes (1.2%) were coded as a psychological symptom diagnosis during the first contact. Most identified patients with psychological symptom diagnoses had only one (n=7608, 72.2%) or two (n=2106, 20.0%) episodes.

### Psychological symptom episodes

**Figure 2:**
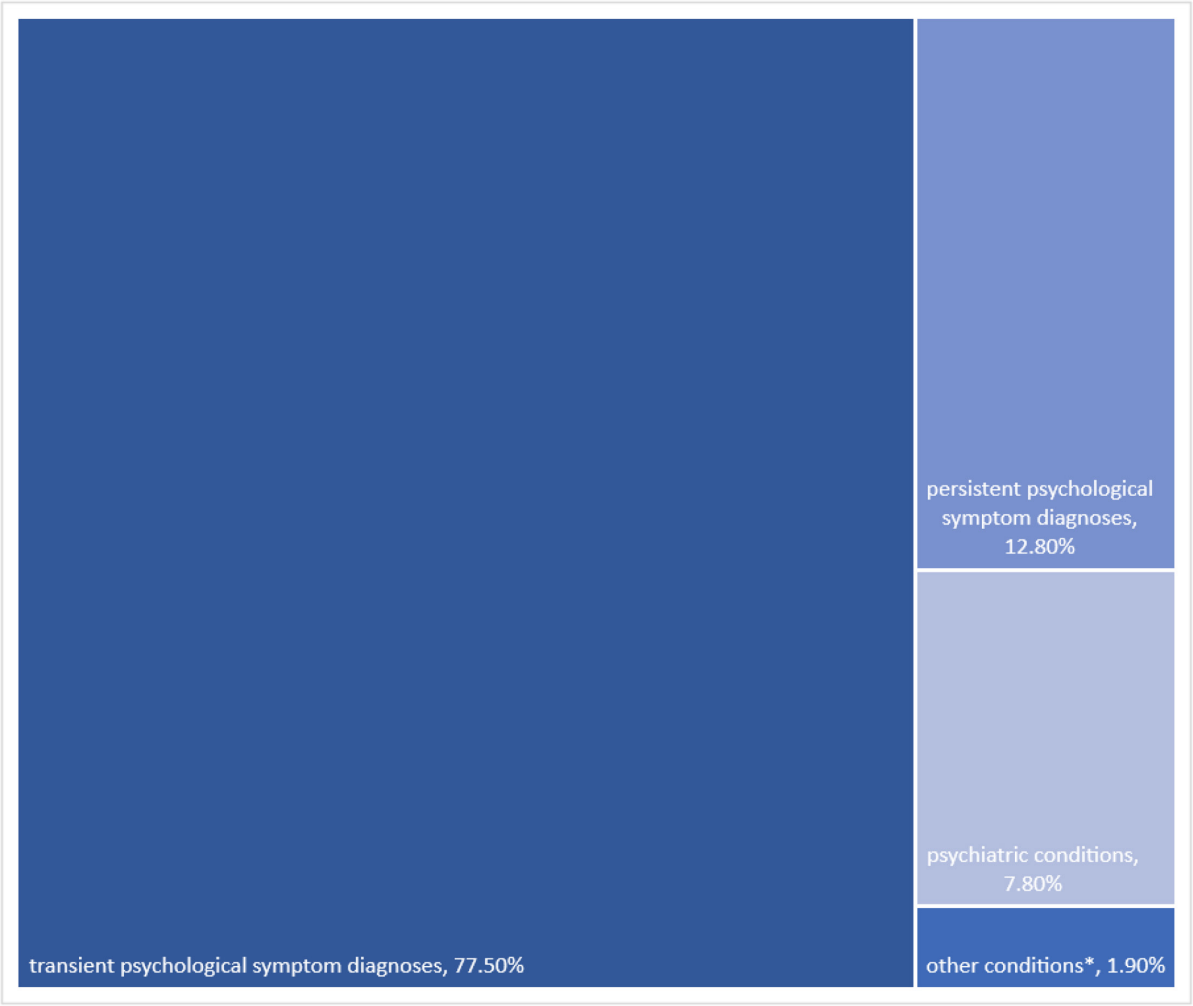
The evolution of psychological symptoms diagnosis after the first contact (n=14 633; 2008–2021).^*^Somatic and social problems, excluded psychological problems, See online supplemental appendix 1 for the list of included psychological symptom diagnosis.

Most of the EoC that started with a psychological symptom diagnosis were transient (n=11 343, 77.5%) with a median number of contacts of 1,^1 2^ 1870 (12.8%) persisted for more than a year with a median number of contacts of 5,^3^,^9^ 1147 (7.8%) changed into psychiatric conditions with a median number of contacts of 8^4^,^16^ and 273 (1.9%) changed into other conditions including somatic and social problems with a median number of contacts of 4^2^,^7^ (figure 2). Among patients who had persistent psychological symptoms, 1211 (64.8%) were females (table 1). Patients having a psychological symptom diagnosis that changed into other conditions such as a somatic condition or a social problem were older (mean=55, SD=21) (table 1).

**Table 1:** Patients’ characteristics and management strategies for episodes that started with a psychological symptom diagnosis (n=14 633; 2008–2021)

	Transient psychological symptom diagnoses	Persistent psychological symptom diagnoses	Psychiatric conditions	Other conditions*
n (%)	11 343 (77.5%)	1870 (12.8%)	1147 (7.8%)	273 (1.9%)
Median duration of the episode of care in days, median (25%–75% pct)	0 (0–34)	938 (576–1633)	545 (166–1290)	98 (8–685)
Sex (females) n (%)	6899 (60.8%)	1211 (64.8%)	702 (61.2%)	169 (61.9%)
Age at the first contact, mean (SD)	45 (19)	48 (18)	43 (21)	55 (21)
Number of contacts with the GP, median, (25%–75% pct)	1 (1–2)	5 (3–9)	8 (4–16)	4 (2–7)
Contacts with mental health nurses (yes) n, %	1181 (10.4%)	376 (20.1%)	264 (23.0%)	19 (7.0%)
Number of interventions, median, (25%–75% pct)	1 (1–3)	5 (3–10)	8 (4–16)	3 (0–6)
Therapeutic counselling/listening (yes) n, %	3434 (30.3%)	771 (41.2%)	584 (50.9%)	77 (28.2%)
Observation/health education/advice (yes) n, %	5648 (49.8%)	1215 (65.0%)	835 (72.8%)	156 (57.1%)
Blood test (yes) n, %	1461 (12.9%)	338 (18.1%)	311 (27.1%)	62 (22.7%)
Number of referrals, median,(25%–75% pct)	0 (0–1)	0 (0–1)	0 (0–1)	0 (0–1)
Referrals to primary care psychologists (yes) n, %	1809 (15.9%)	286 (15.3%)	156 (13.6%)	42 (15.4%)
Referrals to psychotherapists (yes) n, %	232 (2.0%)	33 (1.8%)	22 (1.9%)	2 (0.7%)
Referrals to psychiatrist (yes) n, %	523 (4.6%)	79 (4.2%)	50 (4.4%)	12 (4.4%)
Referrals to mental health institutions (yes) n, %	89 (0.8%)	14 (0.7%)	8 (0.7%)	1 (0.4%)
Number of medication categories, median, (25%–75% pct)	0 (0–1)	0 (0–1)	0 (0–1)	0 (0–1)
Anxiolytics (yes) n, %	1550 (13.7%)	241 (12.9%)	136 (11.9%)	33 (12.1%)
Sedative (yes) n, %	1857 (16.4%)	312 (16.7%)	174 (15.2%)	41 (15.0%)
Antidepressants (yes) n, %	438 (3.9%)	60 (3.2%)	31 (2.7%)	10 (3.7%)
Antipsychotics (yes) n, %	245 (2.2%)	38 (2.0%)	20 (1.7%)	4 (1.5%)
Betablockers (yes) n, %	347 (3.1%)	53 (2.8%)	28 (2.4%)	9 (3.3%)
Top five most common first psychological symptom diagnosis (n, %)				
	Feeling anxious (3193, 28.1%)	Sleep disturbance (785, 42%)	Feeling anxious (278, 24.2%)	Feeling anxious (70, 25.6%)
	Sleep disturbance (2734, 24.1%)	Feeling anxious (404, 21.6%)	Feeling depressed (270, 23.5%)	Memory disturbance (62, 22.7%)
	Feeling depressed (1616, 14.2%)	Feeling depressed (240, 12.8%)	Memory disturbance (220, 19.2%)	Sleep disturbance (38, 22.7%)
	Other psychological symptoms/complaints (1143, 10.1%)	Other psychological symptoms/complaints (146, 7.8%)	Other psychological symptoms/complaints (187, 16.3%)	Other psychological symptoms/complaints (28, 10.3%)
	Acute stress (885, 7.8%)	Memory disturbance (105, 5.6%)	Acute stress reaction (73, 6.4%)	Acute stress reaction (27, 9.9%)

* Other conditions include somatic and social problems, as well as the excluded psychological problems. The full list of included psychological symptom diagnoses is shown in online supplemental appendix 1.

GP, General Practitioner.

Out of the EoC that started with a psychological symptom diagnosis, only 664 (4.5%) were referred to a psychiatrist. Patients with episodes that changed into psychiatric conditions had more contacts with their GPs (median=8, 25/75% pct (4–16)) and more types of interventions (median=8, 25/75% pct (4–16)^16^) compared with patients with episodes that changed into the three other groups (table 1). The median number of medication categories prescribed was 0 (0–1) in all groups. In addition, referrals to primary care psychologists occurred in 15.9% (n=1809) of transient psychological symptoms EoC, 15.3% (n=286) of persistent psychological symptoms EoC and 13.6% (n=156) of psychiatric conditions EoC. Referrals to psychotherapists, psychiatrists and mental health institutions were infrequent and showed minimal variation between groups.

**Figure 3:**
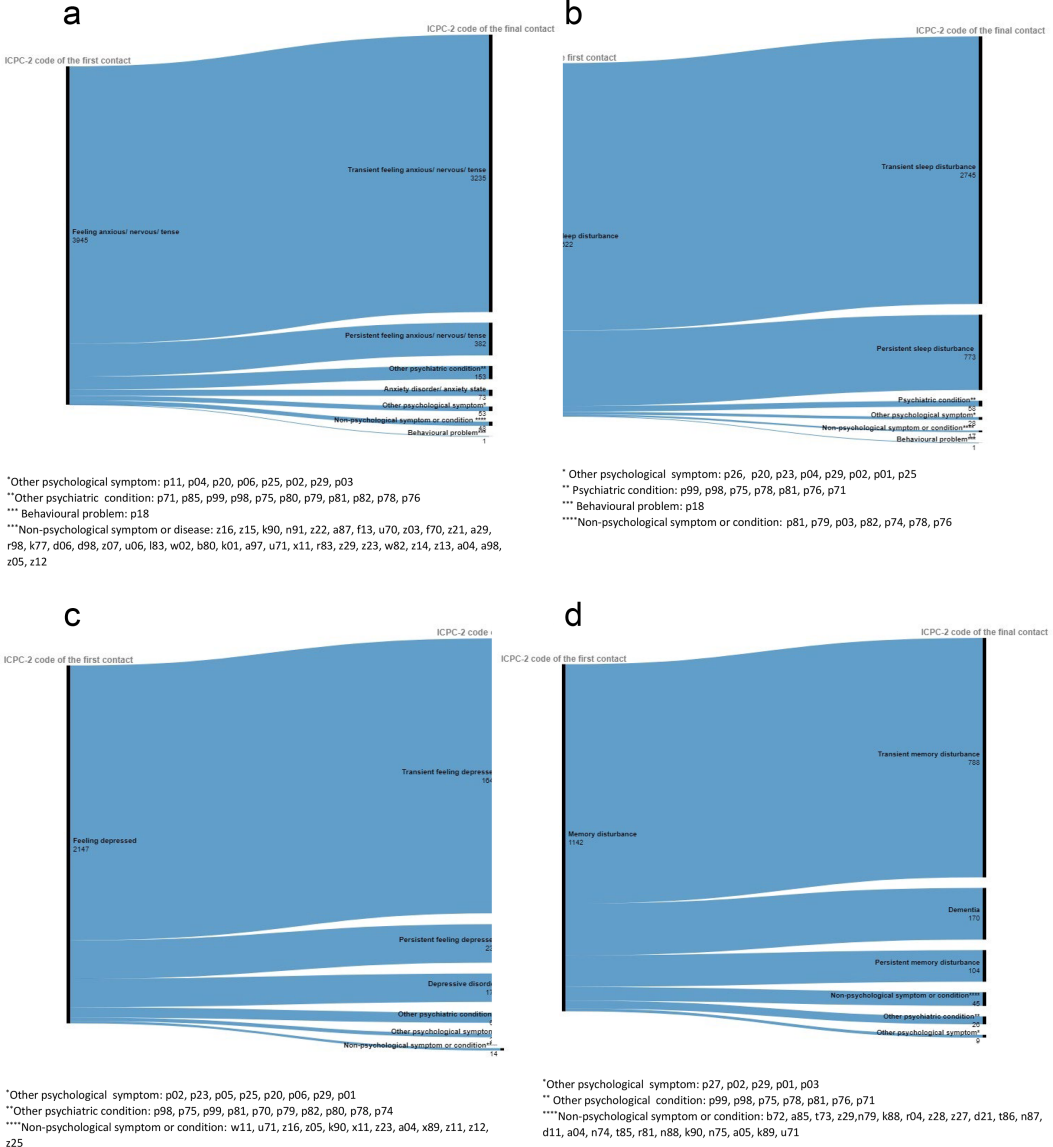
Alluvial diagrams evaluating the course of the most common psychological symptom episodes during the study period 2008–2021 at the first and last contacts, including (**a**) feeling anxious, (**b**) sleep disturbance, (**c**) feeling depressed and (**d**) memory disturbance. ICPC-2, International Classification of Primary Care.

The most common psychological symptom diagnoses after the first contact were “feeling anxious/tense’’, “sleep disturbance’’, “feeling depressed’’, “other psychological symptoms or complaints’’ and “memory disturbance’’. “feeling anxious’’ (figure 3a), “sleep disturbance’’ (figure 3b) and “feeling depressed’’ (figure 3c) evolved more often into persistent psychological symptoms than into psychiatric conditions. However, “Memory disturbance’’ (figure 3d) changed more often into a psychiatric condition, mainly dementia (n=170, 14.9%).

Sleep disturbance was the most prevalent psychological symptom diagnosis, which often evolved into a persistent psychological symptom diagnosis (42%) (table 1).

At the start date of episodes that changed into psychiatric conditions, the most common psychological symptom diagnoses were feeling anxious (24.2%) and feeling depressed (23.5%). Psychological symptom diagnoses that changed into psychiatric conditions most frequently developed into depressive disorders (33.2%), dementia (17.5%) and anxiety disorders (14.9%).

### Comparison of the persistent and psychiatric groups

Patients with persistent psychological symptoms were significantly older (mean=49 years, SD=18) compared with the patients that developed psychiatric conditions (mean=48, SD=21) (table 2).

**Table 2:** Patients’ characteristics and management strategies differences between episodes that persisted as psychological symptom diagnoses and episodes that changed into psychiatric conditions during a 1-year follow-up period during the study period 2008–2021

	Persistent psychological symptom diagnoses (n=1870)	Psychiatric conditions (n=1147)	OR/RR* 95% CI	P value
Sex (females) n (%)	1211 (64.8%)	702 (61.2%)	–	0.052
Age at the first contact, mean (SD)	**49 (18)**	**48 (21)**	–	**<0.001** †
Number of contacts with the GP, mean (SD)	**3.9 (0.05)**	**6.9 (0.08)**	**1.76 (1.63 to 1.91)**	**<0.001** ‡
Contacts with mental health nurses (yes) n, %	**255 (13.6%)**	**192 (16.7%)**	**1.27 (1.04 to 1.56)**	**<0.020** §
Number of interventions, mean (SD)	**7.8 (0.19)**	**13.3 (0.41)**	**1.71 (1.58 to 1.84)**	**<0.001** ‡
Therapeutic counselling/listening (yes) n, %	**593 (31.7%)**	**491 (42.8%)**	**1.61 (1.38 to 1.88)**	**<0.001** §
Observation/health education/advice (yes) n, %	**924 (49.4%)**	**714 (62.2%)**	**1.69 (1.45 to 1.96)**	**<0.001** §
Blood test (yes) n, %	**217 (11.6%)**	**229 (20%)**	**1.90 (1.55 to 2.33)**	**<0.001** §
Number of referrals, mean (SD)	**0.7 (0.03)**	**0.6 (0.03)**	**0.85 (0.75 to 0.95)**	**0.006** ‡
Referrals to primary care psychologists (yes) n, %	286 (15.3%)	156 (13.6%)	0.87 (0.71 to 1.08)	0.202§
Referrals to psychotherapists (yes) n, %	33 (1.8%)	22 (1.9%)	1.09 (0.63 to 1.88)	0.760§
Referrals to psychiatrists (yes) n, %	79 (4.2%)	50 (4.4%)	1.03 (0.72 to 1.48)	0.859§
Referrals to mental health institutions (yes) n, %	14 (0.7%)	8 (0.7%)	0.93 (0.39 to 2.23)	0.873§
Number of medication categories, mean (SD)	1.3 (0.070)	1 (0.089)	0.91 (0.75 to 1.09)	0.293‡
Anxiolytics (yes) n, %	173 (9.3%)	93 (8.1%)	0.87 (0.67 to 1.13)	0.283§
Sedative (yes) n, %	232 (12.4%)	134 (11.7%)	0.93 (0.75 to 1.17)	0.555§
Antidepressants (yes) n, %	51 (2.7%)	24 (2.1%)	0.76 (0.47 to 1.25)	0.278§
Antipsychotics (yes) n, %	26 (1.4%)	8 (0.7%)	0.50 (0.23 to 1.10)	0.086§
Betablockers (yes) n, %	49 (2.6%)	26 (2.3%)	0.86 (0.53 to 1.40)	0.545§

χ2 test.

All significant results are presented in bold (p<0.05).

* Risk ratio (RR) for continuous variables and OR for categorical dichotomous variables; OR and RR reflect episodes that changed into disease diagnoses compared with episodes that persisted as symptom diagnoses.

† Independent T-test.

‡ Negative binomial analysis.

§ Binary logistic regression analysis.

GP, General Practitioner.

Patients who developed psychiatric conditions had a significantly higher number of contacts with the GP during the 1-year follow up (6.9 vs 3.9, RR=1.76, 95% CI 1.63 to 1.91). They showed an increased chance of having at least one contact with a mental health nurse (16.7% vs 13.6%, OR=1.27, 95% CI 1.04 to 1.56). Similarly, this group showed a significantly higher total number of interventions (RR=1.71, 95% CI 1.58 to 1.84) including therapeutic counselling (OR=1.61, 95% CI 1.38 to 1.88, p<0.001), observation/health education or advice (OR=1.69, 95% CI 1.45 to 1.96, p<0.001) and blood tests (OR=1.90, 95% CI 1.55 to 2.33, p<0.001). However, the total number of referrals was significantly lower (RR=0.85, 95% CI 0.75 to 0.95).

## Discussion

This exploratory study shows that, among all health problems, the number of episodes that start with a psychological symptom diagnosis in general practice is low. The large majority of these episodes resolves within a year, less than 15% remain as persistent psychological symptoms and less than 10% change into psychiatric conditions. Out of all episodes that start with a psychological symptom diagnosis, fewer than 5% are referred to a psychiatrist, indicating that most of these episodes are managed in general practice. A significantly higher number of contacts with GPs as well as interventions were observed during the first year of care for episodes that changed into psychiatric conditions compared with episodes that remained as persistent psychological symptoms.

### Comparison with the literature

We found that the frequency of EoC that started with a psychological symptom diagnosis in general practice (1.2%) was lower than the finding in the Danish study (4%).^5^ An explanation of this difference might be different inclusion criteria as the Danish study included all psychological problems including addiction and children’s psychological symptoms, while we have excluded these. In addition, we included only EoCs that started with a contact in which the diagnosis was a psychological symptom, which likely explains the relatively low frequency of these episodes in our dataset. Another explanation might be differences in help-seeking behaviour between both countries, such as the patients’ more frequent access to general practice when facing a psychological or emotional problem in Denmark compared with the Netherlands.^15^

Our finding that the symptom sleep disturbance didn’t persist in 76% of the cases, which is a high percentage compared with the 50% that has been found in patients recruited from an outpatient in an internal medicine clinic in the US.^16^ This difference might be explained by the fact that more severe symptoms, which probably persist more often, might be managed in internal medicine clinics compared with general practice.

As far as we know, there is no publication on management strategies for psychological symptoms in general practice. In our study, we consider the difference in the total number of contacts with the GP and total number of interventions between episodes that developed into psychiatric conditions and those that remained as psychological symptoms as clinically relevant. As the clinical relevance depends not only on differences in numbers or counts, but also on the effect that these differences actually have on the patient’s health, functioning or treatment, we did not consider the differences in the number of contacts with mental health nurses and the total number of referrals as clinically relevant. Although some differences between groups reached statistical significance, their magnitude was small and unlikely to influence clinical decision-making in routine general practice. This assessment was informed by discussions with practising GPs, who confirmed that such differences in the number of contacts with mental health nurses or referrals would not meaningfully affect patient care.

The reform of the Dutch mental health system, introduced in 2014, states that only psychiatric conditions should be referred to professionals working in mental healthcare.^17^ Otherwise, psychological symptom diagnoses with no psychiatric condition labels should be treated in general practice with the support of general practice with more nurse specialists in mental healthcare and additional mental health resources to GPs.^17^ In line with this reform, we could explain our finding that only 5% of all episodes that started with a psychological symptom diagnosis, including episodes that changed into psychiatric conditions, were referred to mental health specialists such as psychiatrists, meaning that most psychological symptoms are managed in primary care settings. The low rate of referrals to mental health specialists for episodes that changed into psychiatric conditions might suggest that these psychiatric conditions are rather mild or moderate within the expertise of primary healthcare providers.^15^ Despite differences in symptom trajectories, the total rates of pharmacological treatment and referrals to psychologists or psychiatrists were surprisingly similar across transient psychological symptoms, persistent psychological symptoms and psychiatric conditions EoC groups. One possible explanation is that many symptom-based episodes represent subthreshold distress or adjustment problems where watchful waiting or non-pharmacological strategies are often preferred. In addition, Dutch GPs may initiate pharmacological treatment without immediately re-labelling an episode as a psychiatric disorder, particularly in situations of diagnostic uncertainty or evolving clinical presentation. Although some degree of diagnostic misclassification cannot be excluded in large databases, the high-quality recording within FaMe-Net suggests that the observed patterns likely reflect genuine similarities in how GPs manage psychological distress in routine care, regardless of the diagnostic label.

The total number of referrals within the first year of care was statistically lower in EoCs that changed into psychiatric conditions compared with persistent psychological symptoms. A possible clinical explanation is that many psychiatric conditions, such as depression or anxiety disorders, are managed pharmacologically by GPs, thereby reducing the need for referral, whereas in patients with persistent psychological symptoms, GPs may still be exploring the underlying cause or most appropriate care pathway. However, the difference in RRs was modest and not clinically relevant.

### Strengths and limitations

As far as we know, this is the first exploratory study that evaluates the course of psychological symptom diagnoses in general practice. The main strength of this study is that we used a PBRN (FaMe-Net), that relies on a population representative of the Dutch population in terms of age and sex, and that is focused on reliable EoC structure that allowed a follow-up of 13 years.^11^

However, our findings should be interpreted taking into account the limitations of the study. First, we might have underestimated the prevalence of psychiatric conditions as our study is based on diagnoses made in everyday practice. Even though GPs are well trained to diagnose psychiatric conditions, the extent of underdiagnosis in this setting remains poorly documented. An old study has suggested that almost half of the patients screened by validated instruments, such as the 12-item General Health Questionnaire,^18^ were not diagnosed by their GPs.^19^ However, while such instruments can help identify possible psychiatric conditions in primary care, they are not definitive diagnostic tools. Second, important aspects such as the severity of the symptoms, level of functioning, quality of life, sociodemographic status, interventions initiated by patients before the first contact with the GP, patient preferences, pre-existing treatment plans and the availability of other information such as the content of letters from medical specialists were not available. Additionally, FaMe-Net GPs may choose to retain the psychological symptom diagnosis, despite a specialist or psychologist diagnosing a psychiatric condition for purposes of administration and reimbursement. These elements are important to assess as they might influence the diagnostic process in general practice as well as the course of symptoms. Furthermore, although FaMe-Net captures the majority of patients’ EoCs, recent transitions, such as changing GP practices, may result in incomplete longitudinal tracking for a small proportion of individuals. Finally, although ICPC is a valuable diagnostic system that allows detailed coding of symptoms, it has some limitations; for example, the category of sleep disturbance can group together both transient complaints and more chronic sleep disorders, potentially obscuring important differences.

### Research and practical implications

In our exploratory study, we found that most psychological symptom episodes were transient and only a small minority changed into psychiatric conditions or persisted as psychological symptoms. These findings seem to indicate that most patients with a psychological symptom diagnosis have a favourable course, although the question remains whether these psychological symptoms actually resolve or persist and the patient does not consider further help desirable or necessary. In our study, we found a significantly higher number of contacts with primary healthcare providers during the first year of care for episodes that changed into psychiatric conditions compared with episodes that remained as persistent psychological symptoms. This might be explained by a higher severity of symptoms in episodes that changed into psychiatric conditions, undertreatment of episodes that persisted as psychological symptoms, or the tendency to over-diagnose psychiatric conditions when patients have more contacts with the GP. Future research should focus on treatment outcomes and success in both groups as well as exploring which patient and GP-related factors contribute to seeking further help from their GPs, whether the symptoms of patients who didn’t seek help, improved and factors that contribute to changes in diagnostic labels from psychological symptoms to psychiatric conditions, in order to gain better insight into prognosis. In addition, we found that most persistent psychological symptoms are treated in primary care settings. However, current Dutch general practice guidelines only cover a few number of psychological symptoms in early stages such as feeling anxious, feeling depressed and sleeping problems.^20^ Therefore, more research on persistent psychological symptom diagnoses is needed to further develop guidelines for a better support of GPs in their practice.

## Supplementary material

10.1136/bmjopen-2025-108541 online supplemental file 1

## Data Availability

Data may be obtained from a third party and are not publicly available.
