# Supplementary material for: Course of psychological symptoms and the initial management strategies in general practice using electronic health records in the Netherlands
Source: BMJ Open. 2026 Apr 21;16(4):e108541. doi: 10.1136/bmjopen-2025-108541 (PMC13110624; doi:10.1136/bmjopen-2025-108541)
Supplement: online supplemental file 1 [file bmjopen-16-4-s001.docx]

Appendices

Appendix 1: The International Classification of Primary Care (ICPC-2) codes of the included psychological symptom diagnosis

|  | ICPC-2 code | Name of the diagnosis |
| --- | --- | --- |
| Psychological symptom diagnoses | P01 | Feeling anxious |
|  | P02 | Acute stress reaction |
|  | P03 | Feeling depressed |
|  | P04 | Feeling/ behaving irritable/ angry |
|  | P05 | Senility, feeling/ behaving old |
|  | P06 | Sleep disturbance |
|  | P07 | Sexual desire reduced |
|  | P08 | Sexual fulfilment reduced |
|  | P09 | Sexual preference concern |
|  | P10 | Stammering/ stuttering/ tic |
|  | P20 | Memory loss |
|  | P27 | Fear of mental disorder |
|  | P28 | Limited function/ disability (P) |
|  | P29 | Psychological symptom/ complt other |

NOS = Not Otherwise Specified.

Appendix 2: List of the included management strategies with corresponding code according to the International Classification of Primary Care (ICPC-2)

| Type of interventions and referrals | ICPC-2 code | Name of interventions |
| --- | --- | --- |
| Type of interventions | -32 | Sensitivity test |
|  | -33 | Microbiological/ immunological test |
|  | -34 | Blood test |
|  | -35 | Urine test |
|  | -36 | Faeces test |
|  | -37 | Histological/ exfoliative cytology |
|  | -38 | Other laboratory test NEC |
|  | -39 | Physical function test |
|  | -40 | Diagnostic endoscopy |
|  | -41 | Diagnostic radiology/ imaging |
|  | -42 | Electrical tracings |
|  | -43 | Other diagnostic procedure |
|  | -44 | Preventive immunizations/ medication |
|  | -45 | Observation/ health education/ advice/ diet |
|  | -45 | Consultation with primary care provider |
|  | -47 | Consultation with specialist |
|  | -48 | Clarification/ discussion of patients rfe/ demand |
|  | -49 | Other preventive procedure |
|  | -51 | Incision/ Drainage/ flushing/ aspiration/ removal body fluid |
|  | -52 | Excision/ removal of tissue/ biopsy/ destruction/debridement |
|  | -53 | Instrumentation/ Catheterization/ intubation/ dilation |
|  | -54 | Repair/ fixation-suture/ cast/ prosthetic device |
|  | -55 | Local injection/ inflation |
|  | -56 | Dressing/ pressure/ compression/ tamponade |
|  | -57 | Physical medicine/ rehabilitation |
|  | -58 | Therapeutic counselling/ listening |
|  | -59 | Other therapeutic procedure NEC |
|  | -60 | Result test/ procedure |
|  | -61 | Result examination/ test/ record/ letter from other provider |
|  | -62 | Administrative procedure |
|  | -63 | Follow-up encounter unspecified |
|  | -64 | Encounter/ problem initiated by provider |
|  | -65 | Encounter/ problem initiated by other than patient/ provider |
| Referrals | -66 | Referrals to other provider (excl. M.D) |
|  | -67 | Referral to physician/ specialist/ clinic/ hospital |

NEC = Not Elsewhere Classified
