# Supplementary material for: Course of psychological symptoms and the initial management strategies in general practice using electronic health records in the Netherlands
Source: BMJ Open. 2026 Apr 21;16(4):e108541. doi: 10.1136/bmjopen-2025-108541 (PMC13110624; doi:10.1136/bmjopen-2025-108541)
Supplement: Supplementary file 2 [file bmjopen-2025-108541.reviewer_comments.pdf]

PEER REVIEW HISTORY

BMJ Open publishes all reviews undertaken for accepted manuscripts. Reviewers are asked to complete a checklist review form and are provided with free text boxes to elaborate on their assessment. These free text comments are reproduced below.

ARTICLE DETAILS

Title (Provisional)

The course of psychological symptoms and the initial management strategies in general practice using electronic health records in the Netherlands

Authors

Chaabouni, Asma; Schouten, Barbara; Houwen, Juul; Lucassen, Peter L.B.J.; Akkermans, Reinier; Walraven, Iris; Van Boven, Kees; Schers, Henk; Olde Hartman, Tim C.

VERSION 1 - REVIEW

**Reviewer**	**1**
**Name**	**Beerten, Simon**
**Affiliation**	**KU Leuven, Department of Public Health and Primary Care**
**Date**	**24-Sep-2025**
**COI**	**None**

General remarks

This paper describes the course and management of psychological symptoms in general practice. While the subject matter is of interest to me, the paper at present is somewhat cluttered and needs additional clarification in the methodology section. Some conclusions do not seem supported by the evidence. The paper would benefit from an in-depth rewriting, more information on methodology and, specifically, the construction of the episode of care.

Major comments

• The Introduction section is confusingly written and lacks direction. The study’s aim and context are only revealed in the second-to-last paragraph of this section. Overall, it should be better structured and the content of the first couple of paragraphs should be more specific and tailored to the aim of the study.

• How is the episode of care structured exactly? From the description it seems as though this is simply first to last contact for a specific health problem. This approach is inherently biased: many intercurrent health problems that are not studied may influence the condition

of the patient leading up to the last contact. As such, I would say it is flawed to observe them as if they existed in a vacuum. Could the authors comment on this?

• If I understand the episode of care in this paper correctly, marking episodes of psychological symptoms as 'transient' makes little sense. Judging from the median of 0 in Table 1, most of these episodes seem to be based on only one consultation/registration. In this case there is no episode of care to construct, because one does not know what happened afterwards: patient seeing a different GP, moving to another region, long-term hospitalization… I would be prudent to draw strong conclusions based on this information. Similarly, how is 'symptom persistence for longer than a year' judged from the episode of care?

• Building on the previous comment, the methodology outlined in the paper also forgoes the possibility of patients presenting with multiple problems at once (both psychological and non-psychological). How did the authors deal with this?

• I am confused by the inclusion of the 'intervention' category. Surely it would seem more logical to only include interventions relevant to psychological problems. There were significantly more interventions in the psychiatric condition group, although the authors do not explain which ones and why this would be.

• Referral rates for the psychiatric condition group were significantly lower. What could be an explanation for this?

Minor comments

• The reported rate of psychological symptoms is low (1.2%), as the authors acknowledge. I do have trouble aligning it with previous work of this group (Chaabouni et al., 2023) where the reported prevalence for all symptom diagnoses is much higher (58%), of which the psychological symptoms seem to be a big part as well. Am I missing something?

• If the Famenet population is representative of the Dutch population in terms of social class, must there not be some variable indicating which 'social class' patients belong to? And if so, why was this variable not included in the regression analyses? This is a strong confounder for studies on uptake of care and referral patterns. Some more information on the database of Famenet in general would also be nice, so readers could compare it to similar national and international databases.

• From a semantic point of view, I would choose another word than 'evolve' for describing the natural progression of symptoms during episodes of care. Now it reads as if some somatic conditions are natural consequences of psychological symptoms.

• Page 11, lines 13-15: the authors state 'we did not consider the differences between (…) as clinically relevant.' This seems a strange statement to me in the middle of the Discussion section. Furthermore, it begs the question: Why?

• Page 12, line 17: '…such instruments are not the gold standards for diagnosing psychiatric conditions.' If validated instruments are not the gold standard, then what is?

• The T test is very sensitive to large sample sizes, so any slight, but not clinically meaningful difference would cause the P value to become 'significant'. Comparing large samples in this way might not be the most informative. It would be better to use a test based on standard deviations (e.g. Cohen's d) if so inclined.

• The reference list needs polishing, particularly when a book or document other than a paper is cited. References 13 and 15 are duplicated.

• Typos:

o Page 7, line 22: '…such as the number of referrals…'

o Page 10, line 59: '…in an internal medicine clinic…'

o Page 11, line 54: '…that relies on a population…"

o Page 13, lines 15-17: '…which factors contribute to…' and '…a psychiatric condition…'

o Figure 2 (caption): '…at the first and last contacts…' and 'b) sleep disturbance'.

**Reviewer**	**2**
**Name**	**Hetlevik, Øystein**
**Affiliation**	**University of Bergen, Department of Global Public Health and Primary Care**
**Date**	**23-Nov-2025**
**COI**	**None**

This article describes the occurrence of episodes in consultations with general practitioners where the patient presents a psychological symptom as the reason for the visit. In addition, it reports on the duration of these episodes and the interventions implemented later in the course. The study is based on data from a research network in the Netherlands where ICPC-2 is used as the coding system for diagnoses.

The article is well-written and clearly structured, with a transparent presentation of data, methods, and results. It is primarily descriptive but includes comparisons of interventions between patients with persistent symptoms and those who eventually receive a psychiatric diagnosis.

It would have been useful to clarify how physicians in routine practice adhere to the definition of "reason for encounter" as described here. On page 4, it is stated that the reason for the encounter is the first thing the patient says to the doctor. While this is a clear definition, is it clinically reasonable? And is it applied so precisely? For example, if a patient says, "I am tired and have a sleep problem," would that be coded as A04, whereas if the

patient says, “I have a sleep problem and feel so tired,” would that be P06? It seems relatively random which word the patient chooses first. This is relevant because the study reports a very low frequency of consultations for psychological problems. Therefore, the selection process should be described in more detail. There may be obvious differences between countries in how general practitioners are perceived as providers of help for mild/common mental health problems.

The course of illness is grouped and clearly described. However, interventions beyond consultations are sparsely presented and discussed, although they appear in the tables. What is not highlighted—but deserves attention and discussion—is the surprisingly similar rates of pharmacological treatment and referrals to psychologists/psychiatrists (other than nurses) across the groups with short-term symptoms, long-term symptoms, and those who eventually receive a psychiatric diagnosis (table 1). Could this indicate that diagnostic coding does not fully reflect the severity of problems? This is addressed to some extent, but these findings could have been emphasised more clearly both in the results and in the discussion.

Among the persistent symptoms, sleep problems are the most frequent. But is this truly a persistent symptom or rather a disease? Keeping this as a symptom is related to a limitation of ICPC as a coding system, where it is not possible to code a sleep disorder, unlike ICD.

One detail: On page 8, line 23—Is “episode” the correct term?

Overall, this is an interesting study, relatively straightforward, based on data from a robust research network, and it is valuable to have this topic illuminated.

VERSION 1 - AUTHOR RESPONSE

Reviewer: 1
Dr. Simon Beerten, KU Leuven
Comments to the Author:
General remarks

This paper describes the course and management of psychological symptoms in general practice. While the subject matter is of interest to me, the paper at present is somewhat cluttered and needs additional clarification in the methodology section. Some conclusions do not seem supported by the evidence. The paper would benefit from an in-depth rewriting, more information on methodology and, specifically, the construction of the episode of care.

Major comments
• The Introduction section is confusingly written and lacks direction. The study’s aim

and context are only revealed in the second-to-last paragraph of this section. Overall, it should be better structured and the content of the first couple of paragraphs should be more specific and tailored to the aim of the study.

We thank the reviewer for this constructive feedback about the introduction. Our initial aim in the first paragraph was to clarify the concept of symptom diagnoses for readers who may not be familiar with it. We agree that the definition part could be more concise and targeted. In response, we removed the following paragraph from the introduction to improve clarity and ensure that the background directly supports the research objectives.

~~*Many patients who experience symptoms do not request medical care. Roughly one in five patients who experience symptoms consult their general practitioner (GP) and about 10% of all symptoms are reported (1, 2). Reasons for patients with a symptom to visit their GP depend on their own perception and interpretation. When presented to a GP, one of the core tasks of the GP is to consider whether the symptom can be attributed to a well-distinguished disease or syndrome. The GP will look also at other causes of symptoms outside the sphere of a disease or a syndrome. Symptoms can be expressions of being aware of physiological functioning or a consequence of sensitisation or somatosensory amplification (3, 4). According to the definition of the world organization of family doctors (WONCA), symptoms are 'any expression of disturbed function or structure of the body or mind by a patient' (5). According to WONCA international classification committee in the International Classification of Primary Care (ICPC), symptom diagnoses are a commonly used diagnostic category to describe a wide range of symptoms that represent the optimal medical label for the health problem (6). Similarly, psychological symptom diagnoses such as feeling anxious, feeling depressed, or being stressed can be part of a psychiatric disorder or can be seen as the consequence of what happens in the patient's life. Theoretically, a psychological symptom can resolve, evolve into a psychiatric disorder, or persist as a psychological symptom diagnosis. The occurrence of symptom diagnoses in general practice is high. A Danish study showed that out of all consultations with patients presenting a health problem, the GP made a symptom diagnosis in 36% of the cases (7). Our study from a large Dutch practice-based research network showed that, out of 1000 patients, around 900 contacted their GP at least once in one year; around 500 contacted their GP for a reason that was diagnosed as a somatic symptom and 75 contacted their GPs for a somatic symptom that persisted for at least one year without evolving into a disease or a syndrome (8). Patients with persistent somatic symptoms reported a high level of burden compared to other patients on the general practice lists (9). Even though a few studies explored the epidemiology of symptom diagnoses, little is known about the course of psychological symptom diagnoses.*~~"

We replaced the paragraph with a more concise and targeted paragraph:

"Patients frequently present psychological complaints, such as feeling anxious or depressed, to their general practitioner (GP). During the consultation, the GP may explore the underlying reasons for these complaints, assess additional symptoms, and determine whether the patient's presentation meets the criteria for a psychiatric disorder (for example, depressive or anxiety disorders). When the symptoms do not fulfil the criteria for a psychiatric disorder, GPs usually label the patient's main complaint as a symptom diagnosis, such as 'feeling depressed' (1-4). In primary care, symptom diagnoses are a commonly used diagnostic category to describe symptoms that do not fulfil the criteria for a disease or disorder. In these circumstances, a symptom diagnosis represents the most appropriate medical label for the health problem (4) During follow-up of patients with a psychological symptom diagnosis, symptoms may resolve, change into a psychiatric condition, or persist as a psychological symptom diagnosis.

The occurrence of symptom diagnoses in general practice is high. A Danish study showed that out of all consultations with patients presenting a health problem, the GP made a symptom diagnosis in 36% of the cases (5). ~~Our study from a large Dutch practice-based research network showed that, out of 1000 patients, around 900 contacted their GP at least once in one year; around 500 contacted their GP for a reason that was diagnosed as a somatic symptom and 75 contacted their GPs for a somatic symptom that persisted for at least one year without evolving into a disease or a syndrome.~~ A Dutch study has shown a higher prevalence (58%) of symptom diagnoses (6). However, these studies only focused on somatic symptom diagnoses, with a lack of information about the course and management of psychological symptom diagnoses. ~~Patients with persistent somatic symptoms reported a high level of burden compared to other patients on the general practice lists (10). Even though a few studies explored the epidemiology of symptom diagnoses, little is known about the course of psychological symptom diagnoses.~~

~~However, there is a scarcity of studies about the management of psychological symptom diagnoses. It is known that~~ This is particularly important in the context of general practice, where consultations for mental health problems are more demanding and tend to take longer (7)."

• How is the episode of care structured exactly? From the description it seems as though this is simply first to last contact for a specific health problem. This approach is inherently biased: many intercurrent health problems that are not studied may influence the condition of the patient leading up to the last contact. As such, I would say it is flawed to observe them as if they existed in a vacuum. Could the authors comment on this?

We understand the reviewer's confusion about the structure of the Episode of Care (EoC). We agree with the reviewer that several intercurrent health problems influence the patient's condition. As such, the structure of the episode of care (EoC) reflects the changes in the medical situation. The system of the EoC is the optimal reflection of daily medical practice. In fact, the GPs in FaMe-Net code the following issues in each contact with the patient: (1) the reason for encounter (the initial presentation of illness - what the patient has said), (2) the diagnosis – the label provided by the GP, (3) the interventions (including diagnostic tests, treatments, and referrals). An EoC refers to a

health problem from the first to the last contact and contains all contacts associated with the initial complaint or reason for encounter. In follow-up consultations, the GP can change the diagnosis in an EoC when new data, for example from additional investigations, become available. It is possible that more than one EoC is registered during a single medical contact depending on the patient's needs and requests for care during that single medical contact. Importantly, GPs register and update diagnoses within each EoC based on their overall clinical assessment, which includes knowledge of comorbidities and intercurrent illnesses. While EoCs are analytically separated, the diagnostic and management decisions recorded within them are made in the context of the patient's full clinical picture. Our analyses therefore reflect how symptom diagnoses are managed in routine practice rather than in isolation.

To ensure high data quality, regular meetings are held among GPs within FaMe-Net to discuss the coding system. Moreover, biannual online 'uniformity' surveys are conducted to promote consistency through training. GPs are trained to register health problems within the EoC framework and to update the coding when symptoms change into defined diseases.

We added the following sentences to the methods to further explain the EoC structure (black: old text, red: new text):

'' To improve reliability of the registration, the FaMe-Net GPs have regular training opportunities, including meetings to discuss the coding system, and biannual online 'uniformity' surveys to promote consistency through training (10). In the Netherlands, GPs act as gatekeepers, and all residents are registered with a single GP practice.''

'All contacts in the FaMe-Net database are registered within an Episode of Care (EoC) structure, defined as a health problem in a patient from the first until the last contact (8). The EoC includes information about the Reason For Encounter (RFE), the diagnosis and the process. The RFE is what the patient first says to the GP, for example ''I'm feeling anxious about my pain'' or ''I want a prescription''. The diagnosis is made by the GP and can be modified during consecutive contacts within the episode. As such, the diagnosis could change from ''feeling anxious'' to an ''anxiety disorder'' over the contacts if the patient gradually fulfils the diagnostic criteria of an ''anxiety disorder''. The database also contains coded information about the process defined as the actions undertaken by the GP: interventions, referrals and treatment including medication (8). Medication is coded following the Anatomical Therapeutic Chemical Classification (ATC) and linked to the EoC. Within FaMe-Net, the EoC structure enables longitudinal tracking of patient information. Changes such as a patient being seen by a different GP within the same practice or experiencing long-term hospitalisation are recorded within the Dutch healthcare system and are, in principle, available to GPs and integrated into the registration system.

During a single medical contact between a patient and a GP, it is possible to record more than one EoC, depending on the patient's needs and requests for care during that single medical contact. EoC diagnoses are coded and updated based on the highest level of diagnostic certainty during medical contacts. Within the FaMe-Net system, multiple EoCs can be active simultaneously. As a result, the course and management of a symptom diagnosis may occur in the context of other ongoing conditions. Importantly, GPs register and update diagnoses within each EoC based on their overall clinical assessment, which includes knowledge of comorbidities and intercurrent illnesses. While EoCs are analytically separated, the diagnostic and management decisions recorded within them are made in the context of the patient's full clinical picture."

• If I understand the episode of care in this paper correctly, marking episodes of psychological symptoms as 'transient' makes little sense. Judging from the median of 0 in Table 1, most of these episodes seem to be based on only one consultation/registration. In this case there is no episode of care to construct, because one does not know what happened afterwards: patient seeing a different GP, moving to another region, long-term hospitalization… I would be prudent to draw strong conclusions based on this information. Similarly, how is 'symptom persistence for longer than a year' judged from the episode of care?

We believe that using Episodes of Care (EoC) is appropriate, as several patients frequently consult their GP only once for a given complaint. This means that after one single contact, there was no follow up with the GP for the same EoC, meaning that the patient did not ask for help for that EoC after one contact. Although it is not always possible to know exactly what happens to the health problem after a single consultation, the EoC structure represents the best available method for capturing the development of illness over time in general practice. In Dutch general practice, patients can only consult another GP within the same practice, which ensures that subsequent contacts are recorded within the same EoC. Moving to another region is relatively uncommon, and most GP practices serve stable populations with long-standing patient–GP relationships. In addition, hospitalisations are recorded within the same system; when a hospital admission is related to the initial complaint, it is registered within the corresponding EoC.

We added the following paragraphs to the methods and limitations respectively:

Methods

In the Netherlands, GPs act as gatekeepers, and all residents are registered with a single GP practice. Within FaMe-Net, the Episode of Care (EoC) structure enables longitudinal tracking of patient information. Changes such as a patient being seen by a different GP within the same practice or experiencing long-term hospitalisation are recorded within

the Dutch healthcare system and are, in principle, available to GPs and integrated into the registration system.

Limitations

Although FaMe-Net captures the majority of patients' EoCs, recent transitions, such as changing GP practices, may result in incomplete longitudinal tracking for a small proportion of individuals.

• Building on the previous comment, the methodology outlined in the paper also forgoes the possibility of patients presenting with multiple problems at once (both psychological and non-psychological). How did the authors deal with this?

We acknowledge that patients in general practice often present multiple concurrent health problems. Within the FaMe-Net system, each health problem is registered in a separate Episode of Care (EoC), and multiple EoCs can be active simultaneously. As a result, the course and management of a symptom diagnosis may occur in the context of other ongoing conditions. Importantly, GPs register and update diagnoses within each EoC based on their overall clinical assessment, which includes knowledge of comorbidities and intercurrent illnesses. While EoCs are analytically separated, the diagnostic and management decisions recorded within them are made in the context of the patient's full clinical picture. Our analyses therefore reflect how symptom diagnoses are managed in routine practice rather than in isolation.

We have added the following sentences to the methods to further clarify this point:

"Within the FaMe-Net system, multiple EoCs can be active simultaneously. As a result, the course and management of a symptom diagnosis may occur in the context of other ongoing conditions. Importantly, GPs register and update diagnoses within each EoC based on their overall clinical assessment, which includes knowledge of comorbidities and intercurrent illnesses. While EoCs are analytically separated, the diagnostic and management decisions recorded within them are made in the context of the patient's full clinical picture.'

• I am confused by the inclusion of the 'intervention' category. Surely it would seem more logical to only include interventions relevant to psychological problems. There were significantly more interventions in the psychiatric condition group, although the authors do not explain which ones and why this would be.

Given the limited existing evidence on how psychological episodes evolve in routine general practice, and recognizing that some episodes initially presenting with psychological symptoms may later develop into somatic conditions, we chose to include all interventions recorded within the same Episode of Care (EoC), following the

International Classification of Primary Care (ICPC-2). We acknowledge the reviewer's point that it might seem more logical to include only interventions directly related to psychological problems and this is exactly what we aimed to achieve by including all interventions related to the same EoC that started with a psychological symptom diagnosis. We believe that including all interventions allows us to capture real-world clinical decision-making and aligns with the exploratory aim of this study.

Overall, the most common interventions were therapeutic counselling/listening, observation/health education/advice, and blood tests, which aligns with expectations in this context of psychological symptoms These interventions were significantly more prevalent in the psychiatric conditions group compared to episodes with persistent or transient psychological symptoms. For example, among episodes starting with a psychological symptom diagnosis (N = 14,633; 2008–2021), therapeutic counselling was provided in 3,434 (30.5%) of transient symptom episodes, 771 (41.2%) of persistent symptom episodes, and 584 (50.9%) of psychiatric condition episodes.

When comparing persistent psychological symptom diagnoses (n = 1,870) with psychiatric conditions (n = 1,147) during the first year of care therapeutic counselling was provided in 593 (31.7%) versus 491 (42.8%) of episodes (OR 1.61, 95% CI 1.38–1.88, $p < 0.001$), observation/health education/advice in 924 (49.4%) versus 714 (62.2%) (OR 1.69, 95% CI 1.45–1.96, $p < 0.001$), and blood tests in 217 (11.6%) versus 229 (20.0%) (OR 1.90, 95% CI 1.55–2.33, $p < 0.001$).

To further explain the intervention category, we added the following sentences to the results section:

'' Similarly, this group showed a significantly higher total number of interventions (RR = 1.71, 95% CI [1.58, 1.84] including therapeutic counselling (OR 1.61, 95% CI 1.38–1.88, $p < 0.001$), observation/health education/advice (OR 1.69, 95% CI 1.45–1.96, $p < 0.001$), and blood tests (OR 1.90, 95% CI 1.55–2.33, $p < 0.001$).''

We also added the following findings to Table 1 and 2 respectively:

Table 1: patients characteristics and management strategies for episodes that started with a symptom diagnosis (N = 14,633; 2008-2021)

	Transient psychological symptom diagnoses	Persistent psychological symptom diagnoses	Psychiatric conditions	Other conditions*
Therapeutic counselling/ Listening (yes) n, %	3434 (30.3%)	771 (41.2%)	584 (50.9%)	77 (28.2%)
Observation/ health education/ advice (yes) n, %	5648 (49.8%)	1215 (65.0%)	835 (72.8%)	156 (57.1%)
Blood test (yes) n, %	1461 (12.9%)	338 (18.1%)	311 (27.1%)	62 (22.7%)

Table 2: Patients characteristics and management strategies differences between episodes that evolved into persistent psychological symptom diagnoses and episodes that evolved into psychiatric conditions during a 1 year follow up period during the study period 2008 - 2021

	Persistent psychological symptom diagnoses (n = 1,870)	Psychiatric conditions (n = 1,147)	OR/RR*95% CI	*p*
**Therapeutic counselling/ Listening (yes) n, %**	**593 (31.7%)**	**491 (42.8%)**	**1.61 [1.38 , 1.88]**	**< 0.001[d]**
**Observation/ health education/ advice (yes) n, %**	**924 (49.4%)**	**714 (62.2%)**	**1.69 [1.45 , 1.96]**	**< 0.001[d]**
**Blood test (yes) n, %**	**217 (11.6%)**	**229 (20%)**	**1.90 [1.55 , 2.33]**	**< 0.001[d]**

[d] binary logistic regression analysis

• Referral rates for the psychiatric condition group were significantly lower. What could be an explanation for this?

The total number of referrals within the first year of care was statistically lower in Episodes of Care (EoCs) that changed into psychiatric conditions compared to those with persistent psychological symptoms (mean: 0.6 vs 0.7; RR = 0.85, 95% CI [0.75, 0.95]). One might initially expect that EoCs evolving into psychiatric conditions would have a higher number of referrals. A possible clinical explanation is that many patients with psychiatric conditions, such as depression or anxiety disorders, are managed pharmacologically by GPs, reducing the need for referral to other services. In contrast, for patients with persistent psychological symptoms, GPs may still be exploring the underlying cause or the most appropriate care pathway, which may result in a higher number of referrals.

Given the large size of the dataset and the small difference in means, these statistically significant differences may not necessarily indicate clinically meaningful differences. Referral patterns in general practice are influenced by multiple factors, including pre-existing treatment plans, interventions initiated by patients before the first contact with the GP, knowledge of comorbidities, context of other conditions and patient preferences, which are not fully captured in the FaMe-Net database. To address this, we added the following sentence to the limitations:

‘’Secondly, important aspects such as the severity of the symptoms, level of functioning, quality of life, socio-demographic status, interventions initiated by patients before the first contact with the GP, patient preferences, pre-existing treatment plans and the availability of other information such as the content of letters from medical specialists were not available.”

Minor comments
• The reported rate of psychological symptoms is low (1.2%), as the authors acknowledge. I do have trouble aligning it with previous work of this group (Chaabouni et al., 2023) where the reported prevalence for all symptom diagnoses is much higher (58%), of which the psychological symptoms seem to be a big part as well. Am I missing something?

In the paper Chaabouni et al., 2023 (8), we identified all patients who had at least one contact with their GP in 2018 and found that 58% of them had at least one symptom diagnosis episode. The definition of symptom diagnosis in (Chaabouni et al., 2023) paper does not include psychological symptoms and only includes somatic symptom diagnoses.

Additionally, the unit of measurement differs between the two studies. In the current paper, we identified EoCs between 2008 and 2021. The median number of patients

included was 30,203, accounting for 1,209,681 EoCs. Of all these episodes, 14,633 (1.2%) were coded as a psychological symptom diagnosis at the first contact.

Thus, the difference in prevalence reflects both the inclusion of psychological symptoms in the current study and the differing denominators: the previous study reported prevalence at the patient level within a single year, whereas the current study reports prevalence at the EoC level across multiple years.

• If the Famenet population is representative of the Dutch population in terms of social class, must there not be some variable indicating which 'social class' patients belong to? And if so, why was this variable not included in the regression analyses? This is a strong confounder for studies on uptake of care and referral patterns. Some more information on the database of Famenet in general would also be nice, so readers could compare it to similar national and international databases.

We thank the reviewer for this important point. In the FaMe-Net database, patient-completed context forms include information on socioeconomic status, employment, and educational status, all of which are approved for use in research. However, these data are only available for around 20% of patients. For these reasons, we focused our study on variables that are consistently available and of high quality in the database. Therefore, it remains unclear to what extent social or contextual factors (such as education, income, employment status, or social support) have influenced the outcome.

• From a semantic point of view, I would choose another word than 'evolve' for describing the natural progression of symptoms during episodes of care. Now it reads as if some somatic conditions are natural consequences of psychological symptoms.

We thank the reviewer for this semantic clarification. Our use of the word "evolve" was intended to describe the longitudinal course or trajectory of psychological symptoms during episodes of care, rather than implying that somatic conditions are a natural consequence of psychological symptoms. To avoid this potential misinterpretation, we have replaced "evolve" with the term ''change'', which more accurately reflects changes in symptom presentation and management over time.

In addition, upon further reflection, we recognized that the term "resolved" could also be confusing, as it may imply confirmed clinical recovery, whereas in this context it reflects the absence of further GP-recorded contacts for that problem. To improve clarity, we have therefore added the following sentence to the methods:

'' For EoC classified as transient, the term "resolved" does not imply confirmed clinical recovery. Rather, it indicates the patient did not seek further help from their GP for that problem.'

• Page 11, lines 13-15: the authors state ‘we did not consider the differences between (...) as clinically relevant.’ This seems a strange statement to me in the middle of the Discussion section. Furthermore, it begs the question: Why?

We thank the reviewer for pointing this out. We agree that the statement could be better contextualized. The intent was to convey that, although some differences between groups reached statistical significance, their magnitude was very small and unlikely to impact clinical decision-making in routine practice. This judgment was informed by discussions with practicing GPs familiar with the management of these conditions, who confirmed that such differences would not meaningfully influence patient care. We have revised the discussion to clarify this rationale and provide context for why these differences were not considered clinically relevant. We added the following sentences to the discussion:

’’As, the clinical relevance depends not only on differences in numbers or counts, but also on the effect that these differences actually have on the patient’s health, functioning, or treatment, we did not consider the differences in the number of contacts with mental health nurses and the total number of referrals as clinically relevant. Although some differences between groups reached statistical significance, their magnitude was small and unlikely to influence clinical decision-making in routine general practice. This assessment was informed by discussions with practicing GPs, who confirmed that such differences in the number of contacts with mental health nurses or referrals would not meaningfully affect patient care.’’

• Page 12, line 17: ‘...such instruments are not the gold standards for diagnosing psychiatric conditions.’ If validated instruments are not the gold standard, then what is?

The instrument we were referring to in this reference was GHQ-12, which is the General Health Questionnaire.

Screening instruments such as the GHQ-12 used in Becker T, Thornicroft G. paper (10) are designed to identify individuals at risk of psychological distress in population or primary care settings, rather than to establish a formal psychiatric diagnosis. By definition, these instruments are not definitive diagnostic tools for psychiatric conditions. For instance, GHD-12 has been criticized for overestimation of its screening accuracy and misclassification of individuals as having psychiatric morbidity when they do not (11). Nevertheless, these instruments could be considered adequate for identifying psychiatric disorders in general practice, as they provide reliable, comparable, and sensitive assessments of psychiatric conditions. To avoid any confusion, we have modified the sentence as follows: *’’Even though some instruments are considered adequate for identifying psychiatric conditions in primary care, such instruments are not definitive diagnostic tools for psychiatric conditions.’’*

• The T test is very sensitive to large sample sizes, so any slight, but not clinically meaningful difference would cause the P value to become 'significant'. Comparing large samples in this way might not be the most informative. It would be better to use a test based on standard deviations (e.g. Cohen's d) if so inclined.

We fully agree with the reviewer. We believe that the best way to assess clinical relevance is through consultation with clinicians as well as by considering the minimum clinically important difference. We previously calculated Cohen's d and found medium effect sizes (d=0.5). Accordingly, we discussed our findings with practicing GPs to understand which differences they would consider meaningful in routine practice. For example, in our study the observed difference of three GP contacts between groups exceeds the minimum clinically important difference reported in the literature, suggesting that this effect is clinically meaningful. However, the difference in the number of referrals within the first year of care between groups was statistically significant (0.7 vs 0.6; RR = 0.85, 95% CI [0.75, 0.95]; p = 0.006), the absolute difference is only 0.1 referrals. Such a small difference is unlikely to have meaningful implications for clinical practice and therefore is not considered clinically relevant. To avoid confusion, we have decided not to describe Cohen's d effect sizes in the manuscript. To further clarify this point, we added the following text to the Discussion in the current version of the manuscript

''Although some differences between groups reached statistical significance, their magnitude was very small and unlikely to influence clinical decision-making in routine general practice. This assessment of the minimum clinically important difference was informed by discussions with practicing GPs, who confirmed that such differences in the number of contacts with mental health nurses or referrals would not meaningfully affect patient care.''

• The reference list needs polishing, particularly when a book or document other than a paper is cited. References 13 and 15 are duplicated.

We have carefully reviewed the reference list and removed the duplicate reference.
• Typos:
o Page 7, line 22: '...such as the number of referrals...'
o Page 10, line 59: '...in an internal medicine clinic...'
o Page 11, line 54: '...that relies on a population..."
o Page 13, lines 15-17: '...which factors contribute to...' and '...a psychiatric condition...'
o Figure 2 (caption): '...at the first and last contacts...' and 'b) sleep disturbance'.

We thank the reviewer for spotting these typos. We have corrected the ones mentioned and conducted a careful review of the entire manuscript to correct additional typos errors as follows:

‘’ Management of patients with psychological symptoms may be especially complex’’

‘’that is focused on reliable EoC structure ~~and~~ that allowed a follow-up of 13 years’’

Reviewer: 2
Prof. Øystein Hetlevik, University of Bergen
Comments to the Author:
This article describes the occurrence of episodes in consultations with general practitioners where the patient presents a psychological symptom as the reason for the visit. In addition, it reports on the duration of these episodes and the interventions implemented later in the course. The study is based on data from a research network in the Netherlands where ICPC-2 is used as the coding system for diagnoses.
The article is well-written and clearly structured, with a transparent presentation of data, methods, and results. It is primarily descriptive but includes comparisons of interventions between patients with persistent symptoms and those who eventually receive a psychiatric diagnosis.

We thank the reviewer for their thoughtful and positive assessment of our manuscript.

It would have been useful to clarify how physicians in routine practice adhere to the definition of “reason for encounter” as described here. On page 4, it is stated that the reason for the encounter is the first thing the patient says to the doctor. While this is a clear definition, is it clinically reasonable? And is it applied so precisely? For example, if a patient says, “I am tired and have a sleep problem,” would that be coded as A04, whereas if the patient says, “I have a sleep problem and feel so tired,” would that be P06? It seems relatively random which word the patient chooses first. This is relevant because the study reports a very low frequency of consultations for psychological problems. Therefore, the selection process should be described in more detail. There may be obvious differences between countries in how general practitioners are perceived as providers of help for mild/common mental health problems.

We thank the reviewer for raising this important point. In the FaMe-Net database, the “reason for encounter” (RFE) is recorded according to ICPC-2 coding guidelines, where the first utterance stated by the patient is coded as the RFE. The RFE reflects the initial presentation of the illness and precedes the interaction between the GP and patient. Previous literature has shown that the RFE has significant predictive value, for example in the diagnosis of cancer (12), supporting the clinical relevance of considering patients’ reasons for encounter.

In the scenario suggested by the reviewer, both “I am tired (A04) and have a sleep problem (P06)” are recorded as RFE of that specific contact, as the FaMe-Net dataset allows the recording of multiple RFEs per contact. Understanding patients’ perceived needs, expectations, and priorities allows GPs to provide individualized care that patients value and that enhances treatment effectiveness. Identifying the RFE also helps GPs address and, when appropriate, influence patients’ health behaviors.

That said, in our study we focused on the diagnosis component of the episode of care (EoC), rather than the RFE. For context [setting paragraph of the methods section], an EoC refers to a health problem from the first to the last contact and is composed of four key elements: (1) RFE, (2) **the diagnosis**, (3) the interventions (including tests, treatments, and referrals), and (4) all contacts associated with the episode. The diagnosis is made by the GP based on clinical judgment. We included only EoCs that started with a contact in which the diagnosis was a psychological symptom, which likely explains the relatively low frequency of these episodes in our dataset.

We added the following sentences (in red) to the discussion to further explain the reasons for this low frequency:

'' We found that the frequency of EoC that started with a psychological symptom diagnosis in general practice (1,2%) was lower than the finding in the Danish study (4%) (7). An explanation of this difference might be different inclusion criteria as the Danish study included all psychological problems including addiction and children's psychological symptoms, while we have excluded these. In addition, we included only EoCs that started with a contact in which the diagnosis was a psychological symptom, which likely explains the relatively low frequency of these episodes in our dataset. Another explanation might be differences in help seeking behaviour between both countries such as the patients' more frequent access to general practice when facing a psychological or emotional problem in Denmark compared to the Netherlands (19).''

The course of illness is grouped and clearly described. However, interventions beyond consultations are sparsely presented and discussed, although they appear in the tables. What is not highlighted—but deserves attention and discussion—is the surprisingly similar rates of pharmacological treatment and referrals to psychologists/psychiatrists (other than nurses) across the groups with short-term symptoms, long-term symptoms, and those who eventually receive a psychiatric diagnosis (table 1). Could this indicate that diagnostic coding does not fully reflect the severity of problems? This is addressed to some extent, but these findings could have been emphasised more clearly both in the results and in the discussion.

We thank the reviewer for highlighting this important observation. The similar rates of pharmacological treatment and referrals across EoC with transient psychological symptoms, persistent psychological symptoms, and psychiatric conditions likely reflect the complexity of primary care management. In routine practice, GPs often initiate similar management strategies based on clinical judgment, patient preferences, and symptom presentation, regardless of whether a formal diagnosis has been established. Management strategies may therefore be guided more by severity, functional impact, and patient preferences than by diagnostic certainty alone. Pharmacological treatment refers specifically to what the GP prescribes within that EoC. Therefore, we agree that

these findings may indicate that diagnostic coding does not always fully capture the severity or complexity of the underlying problems. To address this, we have emphasized these findings more clearly in both the Results and Discussion sections, highlighting that treatment and referral patterns may be influenced by clinical context beyond what is captured in the coded diagnosis.

Results:

"The median number of medication categories prescribed was 0 [0–1] in all groups. In addition, referrals to primary care psychologists occurred in 15.9% (n = 1809) of transient psychological symptoms EoC, 15.3% (n = 286) of persistent psychological symptoms EoC, and 13.6% (n=156) of psychiatric conditions EoC. Referrals to psychotherapists, psychiatrists, and mental health institutions were infrequent and showed minimal variation between groups."

Discussion: Comparison with the literature paragraph

"Despite differences in symptom trajectories, the total rates of pharmacological treatment and referrals to psychologists or psychiatrists were surprisingly similar across transient psychological symptoms, persistent psychological symptoms and psychiatric conditions EoC groups. These findings suggest that both pharmacological and referral-based management were broadly comparable across episodes, regardless of symptom persistence and severity"

Among the persistent symptoms, sleep problems are the most frequent. But is this truly a persistent symptom or rather a disease? Keeping this as a symptom is related to a limitation of ICPC as a coding system, where it is not possible to code a sleep disorder, unlike ICD.

We thank the reviewer for this important observation. Within ICPC-2, sleep problems are primarily coded at the symptom level (P06), which may group together transient complaints and more chronic sleep disorders. These are recorded as a symptom rather than a disorder, which differs from ICD coding where sleep disorders can be classified as distinct disorders. We therefore reported sleep problems as symptoms, in line with the ICPC-2 coding structure. This classification feature may have contributed to the relatively high proportion of persistent sleep-related symptom diagnoses in our study. We added the following limitation to the discussion

"Finally, although ICPC is a valuable diagnostic system that allows detailed coding of symptoms, it has some limitations, for example, the category of sleep disturbance can group together both transient complaints and more chronic sleep disorders, potentially obscuring important differences."

One detail: On page 8, line 23—Is “episode” the correct term?

The reviewer is referring to the following sentence: ‘’*we counted the number of contacts during each* ***episode*** *for face-to-face consultations, out-of-hours services, telephone and e-consultations*.” We actually mean an EoC and have changed the term episode with EoC in the sentence as follows to clarify any confusion.

“We counted the number of contacts during each ***EoC*** *for face-to-face consultations, out-of-hours services, telephone and e-consultations.”*

Overall, this is an interesting study, relatively straightforward, based on data from a robust research network, and it is valuable to have this topic illuminated.

References

1. Green LA, Fryer GE, Jr., Yawn BP, Lanier D, Dovey SM. The ecology of medical care revisited. N Engl J Med. 2001;344(26):2021-5.
2. van de Lisdonk EH. Perceived and presented morbidity in general practice. A study with diaries in four general practices in The Netherlands. Scand J Prim Health Care. 1989;7(2):73-8.
3. Rosendal M, Hartman TCO, Aamland A, van der Horst H, Lucassen P, Budtz-Lilly A, et al. "Medically unexplained" symptoms and symptom disorders in primary care: prognosis-based recognition and classification. Bmc Family Practice. 2017;18.
4. Deary V, Chalder T, Sharpe M. The cognitive behavioural model of medically unexplained symptoms: a theoretical and empirical review. Clin Psychol Rev. 2007;27(7):781-97.
5. Malterud K, Guassora AD, Graungaard AH, Reventlow S. Understanding medical symptoms: a conceptual review and analysis. Theor Med Bioeth. 2015;36(6):411-24.
6. . WONCA INTERNATIONAL DICTIONARY FOR GENERAL/FAMILY PRACTICE.
7. Rosendal M, Carlsen AH, Rask MT, Moth G. Symptoms as the main problem in primary care: A cross-sectional study of frequency and characteristics. Scand J Prim Health. 2015;33(2):91-9.
8. Chaabouni A, Houwen J, Walraven I, van Boven K, Peters H, Schers H, et al. Patients' Characteristics and General Practitioners' Management of Patients with Symptom Diagnoses. J Am Board Fam Med. 2023;36(3):477-92.
9. Chaabouni A, Houwen J, Grewer G, Liebau M, Akkermans R, van Boven K, et al. The burden of persistent symptom diagnoses in primary care patients: a cross-sectional study. Scand J Prim Health Care. 2024:1-11.
10. Becker T, Thornicroft G. Mental illness in general health care. An international study - Ustun,TB, Sartorius,N. Int J Geriatr Psych. 1996;11(11):1021-.
11. Hankins M. The reliability of the twelve-item general health questionnaire (GHQ-12) under realistic assumptions. BMC Public Health. 2008;8:355.

12. van Boven K, Uijen AA, van de Wiel N, Oskam SK, Schers HJ, Assendelft WJJ. The Diagnostic Value of the Patient's Reason for Encounter for Diagnosing Cancer in Primary Care. J Am Board Fam Med. 2017;30(6):806-12.

VERSION 2 - REVIEW

Reviewer **1**

Name **Beerten, Simon**

Affiliation **KU Leuven, Department of Public Health and Primary Care**

Date **22-Feb-2026**

COI

I thank the authors for the extensive revision of their paper and the detailed responses to my questions. The clarity and transparency of the manuscript have greatly improved, specifically regarding the use of the 'episode of care' construct.

There are still some things I would like to highlight:

- In response to my question on referral rates (p. 7 of the rebuttal document), the authors provide a possible explanation (pharmacological management). This is not present in the revised manuscript, but I would urge the authors to include it.

- Please cite the GHQ-12 in the revised manuscript, as this appeared to be the instrument the authors were referring to, as discussed on p. 15 of the rebuttal.

- In my view, the fact that only 20% of the study population has SES descriptors does not justify excluding this variable from the analysis. Including some sort of sensitivity analysis with this variable would make the paper even more transparent and interesting. If those descriptors are really of low quality, as the authors seem to imply, then that should be made more explicit in the manuscript.

The paper has improved enough for me to be content with it. I therefore leave it to the Editor to decide if additional SES analyses are needed and would add value. In case the authors proceed with these analyses, I would like to see the relevant revised portion of the manuscript again. Else, I do not need to see the manuscript again.

Reviewer **2**

Name **Hetlevik, Øystein**

Affiliation **University of Bergen, Department of Global Public Health and Primary Care**

**Date**	**22-Feb-2026**
**COI**	

Thank you for this new version, with very clear and comprehensive responses to comments.

I realised that I had given comments to the first submission based on the misunderstanding that the “diagnosis” from Reason for encounter was the first registration within an EoC. That was a misreading from me, related to the difficulty of fully understanding the “Duch way” of registering data, but also supported by the authors’ focus on RFE in the method. Why are the RFE presented at all, as these data have no relevance for this study?

Further, I still wonder about the small differences between the GPs' handling of patients with persistent symptoms diagnosis compared to those with a psychiatric condition. I surely agree with the comments from the authors that GP practice is complex, but I cannot see this as a full explanation of this paradox. An example related to depression: In the psychiatric conditions group, one third had depression, and if all those were treated with antidepressants, that would constitute approx 6 %. This contrasts very clearly with a study based on another research network that shows nearly 10 times as many patients were treated with antidepressants in a new episode of depression *. These studies are not quite comparable, especially if a majority of patients are diagnosed with a psychiatric condition as the first diagnosis in Duch general practice, but the differences are surprising. It would have helped to understand the context if information had been provided on the proportion of episodes of care that start with the diagnosis of a psychiatric condition, to compare to the numbers for symptoms used in this study, if available. There may be differences in the use of specific disease diagnoses across cultures.

However, could there also be misclassification since there seem to be a lot of patients with “only” symptoms who had antidepressants, i.e.? This brings up an alternative explanation, that the doctors are not precise in coding diagnoses and changing the EoC codes. The new sentence in the discussion, after describing the similarities: “These findings suggest that both pharmacological and referral-based management are broadly comparable across episodes, regardless of symptoms persistence and severity “ is vague and does not reflect any interpretation of this surprising result. That should have been elaborated on since the precision in the use of ICPC codes in the EoC structures is the foundation of this study.

*Hansen, A.B., Baste, V., Hetlevik, Ø. et al. Comparison of depression care provided in general practice in Norway and the Netherlands: registry-based cohort study (The Norwegian GP-DEP study). BMC Health Serv Res 22, 1494 (2022). https://doi.org/10.1186/s12913-022-08793-7

VERSION 2 - AUTHOR RESPONSE

n/a

Reviewer: 1
Dr. Simon Beerten, KU Leuven
Comments to the Author:
I thank the authors for the extensive revision of their paper and the detailed responses to my questions. The clarity and transparency of the manuscript have greatly improved, specifically regarding the use of the 'episode of care' construct.

There are still some things I would like to highlight:
- In response to my question on referral rates (p. 7 of the rebuttal document), the authors provide a possible explanation (pharmacological management). This is not present in the revised manuscript, but I would urge the authors to include it.

The reviewer is referring to the following explanation about the significantly lower referral rates in the psychiatric conditions group: '' The total number of referrals within the first year of care was statistically lower in Episodes of Care (EoCs) that changed into psychiatric conditions compared to those with persistent psychological symptoms (mean: 0.6 vs 0.7; RR = 0.85, 95% CI [0.75, 0.95]). One might initially expect that EoCs evolving into psychiatric conditions would have a higher number of referrals. A possible clinical explanation is that patients who are diagnosed by the GP with a psychiatric condition, such as a depression or an anxiety disorder, are " clear cases" and therefore are managed (e.g. pharmacologically) by the GP, reducing the need for referral to other services. In contrast, for patients who end up with persistent psychological symptoms, GPs may have a diagnostic challenge, sometimes resulting in higher number of referrals, in order to explore the underlying cause or the most appropriate care pathway. However, the difference in rate ratios is moderate (15% relative reduction in outcome rate) and not clinically relevant.

We added the following sentences to the discussion

''The total number of referrals within the first year of care was statistically lower in EoCs that changed into psychiatric conditions compared with persistent psychological symptoms. A possible clinical explanation is that many psychiatric conditions, such as depression or anxiety disorders, are managed pharmacologically by GPs, thereby reducing the need for referral, whereas in patients with persistent psychological symptoms GPs may still be exploring the underlying cause or most appropriate care pathway. However, the difference in rate ratios was modest and not clinically relevant.''

- Please cite the GHQ-12 in the revised manuscript, as this appeared to be the instrument the authors were referring to, as discussed on p. 15 of the rebuttal.

We have added the following citation to this sentence in the limitations

'' An old study showed that almost half of the patients screened by validated instruments, such as 12-item General Health Questionnaire [20], were not diagnosed by their GPs [18]''

References

20. Goldberg, D., *General health questionnaire-12.* Australian Journal of Psychology, 1978.

- In my view, the fact that only 20% of the study population has SES descriptors does not justify excluding this variable from the analysis. Including some sort of sensitivity analysis with this variable would make the paper even more transparent and interesting. If those descriptors are really of low quality, as the authors seem to imply, then that should be made more explicit in the manuscript.

SES indicators appeared to be recorded for only approximately 20% of patients in the FaMe-Net database and were not collected systematically but depended on practice-level administrative routines. Exploratory analyses from one of our studies have showed that patients with available SES data differed systematically from patients without these data suggesting that the missingness is not random but informative. For example, patients with SES data were significantly younger (89.7% aged <65 years vs 53.9%). As a result, the available SES data represent a selective subgroup rather than a representative subset of the cohort. Because the missingness is likely informative, including SES in analyses would risk introducing bias rather than reducing confounding.

For transparency, we have added the following sentence to the methods, data collection, demographics paragraph

"More socioeconomic variables were available through forms filled in by patients. However, these data were available for only approximately 20% of the study population and were considered insufficiently representative to be included in the main analyses. Exploratory analyses showed that patients with available SES data differed systematically from those without these data (e.g., they were significantly younger), suggesting potential selection bias. Therefore, these variables were not incorporated into the analyses."

The paper has improved enough for me to be content with it. I therefore leave it to the Editor to decide if additional SES analyses are needed and would add value. In case the authors proceed with these analyses, I would like to see the relevant revised portion of the manuscript again. Else, I do not need to see the manuscript again.

Reviewer: 2
Prof. Øystein Hetlevik, University of Bergen
Comments to the Author:
Thank you for this new version, with very clear and comprehensive responses to comments.

I realised that I had given comments to the first submission based on the misunderstanding that the "diagnosis" from Reason for encounter was the first registration within an EoC. That was a misreading from me, related to the difficulty of fully understanding the "Duch way" of registering data, but also supported by the authors' focus on RFE in the method. Why are the RFE presented at all, as these data have no relevance for this study?

We agree that the inclusion of RFE in earlier versions may have contributed to confusion. RFE data were not used analytically but were originally described to clarify the Dutch episode-of-care structure.

We shortened the section about the RFE and deleted the following sentence

~~*''The RFE is what the patient first says to the GP, for example ''I'm feeling anxious about my pain'' or ''I want a prescription''.*~~

Further, I still wonder about the small differences between the GPs' handling of patients with persistent symptoms diagnosis compared to those with a psychiatric condition. I surely agree with the comments from the authors that GP practice is complex, but I cannot see this as a full explanation of this paradox. An example related to depression: In the psychiatric conditions group, one third had depression, and if all those were treated with antidepressants, that would constitute approx 6 %. This contrasts very clearly with a study based on another research network that shows nearly 10 times as many patients were treated with antidepressants in a new episode of depression *. These studies are not quite comparable, especially if a majority of patients are diagnosed with a psychiatric condition as the first diagnosis in Duch general practice, but the differences are surprising. It would have helped to understand the context if information had been provided on the proportion of episodes of care that start with the diagnosis of a psychiatric condition, to compare to the numbers for symptoms used in this study, if available. There may be differences in the use of specific disease diagnoses across cultures.
However, could there also be misclassification since there seem to be a lot of patients with "only" symptoms who had antidepressants, i.e.? This brings up an alternative explanation, that the doctors are not precise in coding diagnoses and changing the EoC codes. The new sentence in the discussion, after describing the similarities: "These findings suggest that both pharmacological and referral-based management are broadly comparable across episodes, regardless of symptoms persistence and severity " is vague and does not reflect any interpretation of this surprising result. That should have been elaborated on since the precision in the use of ICPC codes in the EoC structures is the foundation of this study.

*Hansen, A.B., Baste, V., Hetlevik, Ø. et al. Comparison of depression care provided in general practice in Norway and the Netherlands: registry-based cohort study (The

Norwegian GP-DEP study). BMC Health Serv Res 22, 1494 (2022).
https://doi.org/10.1186/s12913-022-08793-7

We appreciate the reviewer's thoughtful comparison with the study by Hansen, A.B. et al. and the opportunity to clarify this important point. We agree that the relatively small differences in management between patients with persistent symptom diagnoses and those with psychiatric conditions warrant careful interpretation.

The study by Hansen, A.B. et al. included patients with newly diagnosed depressive disorder (P76) [1], which is a psychiatric condition following the International Classification of Primary Care (ICPC), However, our cohort consisted of episodes initiated with psychological symptom diagnoses rather than established psychiatric condition codes. This distinction is crucial. In Dutch general practice, many depressive episodes may start directly with a psychiatric diagnosis and would therefore not be captured within our symptom-based cohort. As such, the denominator and clinical context differ substantially between the two studies, which limits direct comparability.

The relatively modest antidepressant use observed in our cohort likely reflects that many symptom-based episodes represent subthreshold distress or adjustment problems, where watchful waiting or non-pharmacological strategies are often preferred. In addition, Dutch GPs may initiate pharmacological treatment without immediately re-labelling an episode as a psychiatric disorder, particularly in situations of diagnostic uncertainty or evolving clinical presentation.

We agree that coding practices deserve explicit consideration in all routinely collected healthcare databases. For example, in the Nivel Primary Care Database used by Hansen AB et al., discrepancies between prescribed and actually dispensed medication have been reported, potentially leading to overestimation of actual drug exposure [2]. Another example comes from our own previous work with the AHON Database, a Dutch registry with a similar coding structure to NIVEL, which comprises a much larger number of general practices (around 90 practices and >460,000 patients) with routinely collected electronic health record data primarily intended for local research and quality reporting. In a pilot study analyzing diagnostic coding and free-text entries, we found that only approximately 50% of the diagnoses were coded correctly. All of these findings reflect how misclassification is an inherent limitation in routine primary care databases and emphasize the importance of careful interpretation of prescribing and diagnostic data when conducting epidemiological analyses [3]. In contrast, the FaMe-Net uses a structured, episode-based registration system in which prescriptions are recorded as part of a validated longitudinal episode of care, consistently linked to the relevant diagnosis and management decision, reducing the risk of misclassification in analyses of prescribing and treatment patterns. Although some degree of diagnostic misclassification cannot be excluded, we believe that our findings mostly reflect

genuine similarities in how GPs manage psychological distress in routine care, regardless of the diagnostic label.

We have elaborated on these issues in the Discussion to provide a more explicit interpretation of this finding.

‘’Despite differences in symptom trajectories, the total rates of pharmacological treatment and referrals to psychologists or psychiatrists were surprisingly similar across transient psychological symptoms, persistent psychological symptoms and psychiatric conditions EoC groups. ~~These findings suggest that both pharmacological and referral-based management were broadly comparable across episodes, regardless of symptom persistence and severity.~~ One possible explanation is that many symptom-based episodes represent subthreshold distress or adjustment problems where watchful waiting or non-pharmacological strategies are often preferred. In addition, Dutch GPs may initiate pharmacological treatment without immediately re-labelling an episode as a psychiatric disorder, particularly in situations of diagnostic uncertainty or evolving clinical presentation. Although some degree of diagnostic misclassification cannot be excluded in large databases, the high-quality recording within FaMe-Net suggests that the observed patterns likely reflect genuine similarities in how GPs manage psychological distress in routine care, regardless of the diagnostic label.”

References

1. Hansen, A.B., et al., *Comparison of depression care provided in general practice in Norway and the Netherlands: registry-based cohort study (The Norwegian GP-DEP study).* BMC Health Serv Res, 2022. **22**(1): p. 1494.
2. Hempenius, M., et al., *Primary nonadherence to drugs prescribed by general practitioners: A Dutch database study.* Br J Clin Pharmacol, 2023. **89**(1): p. 268-278.
3. Chaabouni, A., *The epidemiology of symptom diagnoses in general practice (Doctoral dissertation).* https://repository.ubn.ru.nl/bitstream/handle/2066/318152/318152.pdf?isAllowed=y&sequence=1, 2025.

VERSION 3 - REVIEW

Reviewer 2

Name Hetlevik, Øystein

Affiliation University of Bergen, Department of Global Public Health and Primary Care

Date 16-Mar-2026

COI

Thank you for the thorough discussion of my previous comments. The revised version addresses the potential weaknesses in a clear and satisfactory manner, and I have no further comments.
